# PARP-1: a critical regulator in radioprotection and radiotherapy-mechanisms, challenges, and therapeutic opportunities

**DOI:** 10.3389/fphar.2023.1198948

**Published:** 2023-06-06

**Authors:** Wen-Hao Li, Fei Wang, Gui-Yuan Song, Qing-Hua Yu, Rui-Peng Du, Ping Xu

**Affiliations:** ^1^ School of Food and Biomedicine, Zaozhuang University, Zaozhuang, Shandong, China; ^2^ School of Public Health, Weifang Medical University, Weifang, Shandong, China

**Keywords:** ionizing radiation, PARP inhibitor, p53/ROS, NF-κB/DNA-PK, Caspese-3/AIF

## Abstract

**Background:** Since its discovery, poly (ADP-ribose) polymerase 1 (PARP-1) has been extensively studied due to its regulatory role in numerous biologically crucial pathways. PARP inhibitors have opened new therapeutic avenues for cancer patients and have gained approval as standalone treatments for certain types of cancer. With continued advancements in the research of PARP inhibitors, we can fully realize their potential as therapeutic targets for various diseases.

**Purpose:** To assess the current understanding of PARP-1 mechanisms in radioprotection and radiotherapy based on the literature.

**Methods:** We searched the PubMed database and summarized information on PARP inhibitors, the interaction of PARP-1 with DNA, and the relationships between PARP-1 and p53/ROS, NF-κB/DNA-PK, and caspase3/AIF, respectively.

**Results:** The enzyme PARP-1 plays a crucial role in repairing DNA damage and modifying proteins. Cells exposed to radiation can experience DNA damage, such as single-, intra-, or inter-strand damage. This damage, associated with replication fork stagnation, triggers DNA repair mechanisms, including those involving PARP-1. The activity of PARP-1 increases 500-fold on DNA binding. Studies on PARP-1-knockdown mice have shown that the protein regulates the response to radiation. A lack of PARP-1 also increases the organism’s sensitivity to radiation injury. PARP-1 has been found positively or negatively regulate the expression of specific genes through its modulation of key transcription factors and other molecules, including NF-κB, p53, Caspase 3, reactive oxygen species (ROS), and apoptosis-inducing factor (AIF).

**Conclusion:** This review provides a comprehensive analysis of the physiological and pathological roles of PARP-1 and examines the impact of PARP-1 inhibitors under conditions of ionizing radiation exposure. The review also emphasizes the challenges and opportunities for developing PARP-1 inhibitors to improve the clinical outcomes of ionizing radiation damage.

## 1 Introduction

**FIGURE 1:**
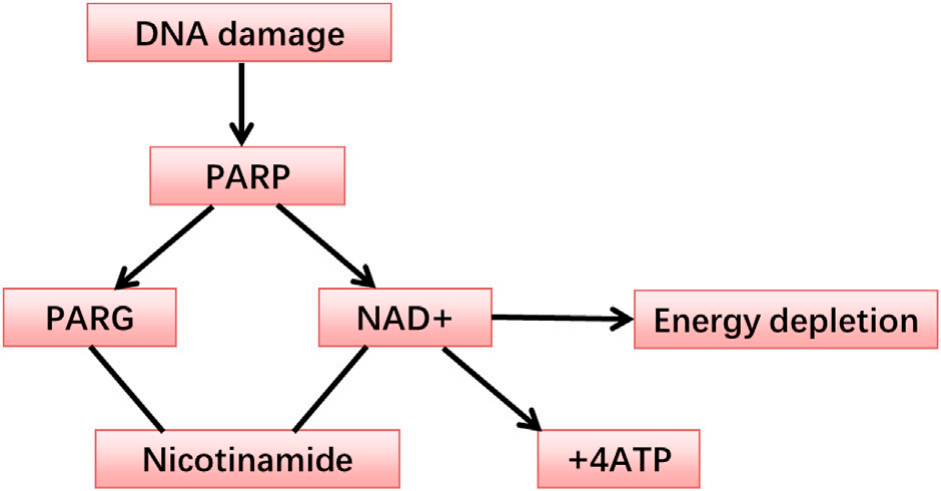
The metabolism of the PARP.

**FIGURE 2:**
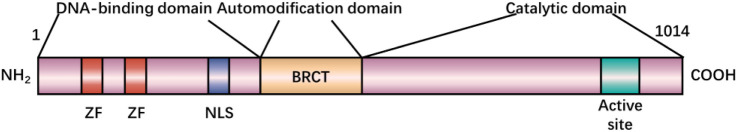
Primary structure of poly(ADP-ribose) polymerase 1 (PARP-1).

The PARP-1 nucleoprotein is expressed at high levels in eukaryotic cells, where it serves as an essential component of the DNA base excision-repair (BER) system (Demin et al., 2021). Recent evidence has also shown that PARP-1 plays roles in nucleotide excision repair (NER) (Pines et al., 2012; Robu et al., 2013), classical non-homologous end joining (cNHEJ) (Ruscetti et al., 1998; Spagnolo et al., 2012; Luijsterburg et al., 2016), alternative NHEJ (aNHEJ) (Wang et al., 2006; Fattah et al., 2010; Mansour et al., 2010; Cheng et al., 2011; Sfeir and de Lange., 2012; Ceccaldi et al., 2015; Mateos-Gomez et al., 2015), microhomology-mediated end-joining (MMEJ) (Sfeir and Symington, 2015; Dutta et al., 2017), homologous recombination (HR) (Hochegger et al., 2006; Hu et al., 2014), DNA mismatch repair (MMR) (Liu et al., 2011), and maintenance of replication fork stability (Yang et al., 2004; Bryant et al., 2009; Ray Chaudhuri et al., 2012; Berti et al., 2013; Ronson et al., 2018) pathways. Two recent reviews have explored how PARP-1 functions in these different pathways (Martin-Hernandez et al., 2017; Ray Chaudhuri and Nussenzweig, 2017). In addition to its role as a mediator of DNA repair, PARP-1 also functions as a regulator of transcription, post-transcriptional gene expression, cell death, and inflammatory activity (Rajawat et al., 2017). PARP-1 belongs to a family of enzymes that catalyze the transfer of ADP-ribose from the substrate, nicotinamide adenine dinucleotide (NAD), to particular nucleoprotein targets (Figure 1). Functionally, PARP-1 acts as a sensor for gaps in DNA and can interact with various DNA repair factors in the BER pathway (Muiras, 2003). PARP-1 contains several conserved and functionally distinct domains, including a self-modifying domain, a 55-kDa catalytic domain, and two zinc finger domains that bind DNA (Kotova et al., 2010). PARP-1 is mobilized to the locations of DNA damage. This mobilization results in the two homologous N-terminal PARP-1 zinc finger domains binding to the compromised DNA, consequently leading to a 500-fold enhancement in the enzymatic activity of PARP-1 (Langelier et al., 2008; Tao et al., 2008). A third zinc finger domain is specific to PARP-1 and essential for its DNA-dependent activity (Gamsjaeger et al., 2007). The structure of PARP-1 is shown in Figure 2.

PARP-1 inhibitors can be used in combination with ionizing radiation or chemotherapy to enhance the sensitivity of tumor cells to these treatments. They can also serve as standalone treatments that are both effective and relatively non-toxic for cancers with DNA repair gene defects, including those with BRCA-1/2 mutations, such as some triple-negative breast cancers. Most PARP inhibitors developed to date are not specific for PARP-1 and function by interacting with the catalytic NAD-binding domain and thus competing with NAD for PARP-1 binding by simulating the substrate-enzyme interaction and interfering with double-stranded break (DSB) repair (Langelier et al., 2018). Others inhibit PARP-1 allosterically by binding to the catalytic domain, impairing the DNA-binding ability and, thus, the DSB repair response (Ogden et al., 2021; Rouleau-Turcotte et al., 2022).

The transcription factor NF-κB, associated with inflammation, is typically found in the cytosol bound to the inhibitory protein IκB. When stimulated by intracellular or extracellular stimuli, IκB is degraded, leading to the nuclear translocation of NF-κB, which binds to the promoter regions of specific target genes, upregulating their expression (Perkins, 2000; Negi and Sharma, 2015). PARP-1 can form a complex with NF-κB to promote NF-κB target gene expression (Ullrich et al., 2001). Both NF-κB DNA-binding activity and induced nitric oxide synthase (iNOS) expression in macrophages were found to be reduced by PARP-1 inhibitors (Chiarugi, 2002). PARP-knockdown mice exposed to endotoxin-induced shock also exhibited impaired NF-κB activation and iNOS upregulation (Oliver et al., 1999). Indeed, PARP-1 and NF-κB are closely associated with one another, with synthesized PAR facilitating the trans-stimulation of NF-κB (Smith, 2001), enabling PARP-1 to regulate NF-κB activity (Hunter et al., 2012). NF-κB activation is important for p53 expression and activity, and PARP can thus potentially shape cell fate outcomes by regulating p53, NF-κB, and a range of different effector proteins (Qin et al., 1999; Ryan et al., 2000). PAR-binding sequence motifs exist in many proteins, including NF-κB, p53, and DNA-dependent protein kinase (DNA-PK) (Pleschke et al., 2000). The PAR-binding zinc finger (PBZ) motif in several eukaryotic proteins is involved in the DNA damage response and checkpoint regulation (Ahel et al., 2008; Eustermann et al., 2010).

Cell death facilitated by PARP-1 shares many features with typical apoptosis, necessitating the relocation of the mitochondrial AIF-1 to the nuclear compartment (Lautier et al., 1993; Alano et al., 2004). Sphingosine and radiation can enhance caspase-independent apoptotic death in radioresistant Jurkat T cell clones, demonstrating a role for PARP-1 activation in AIF translocation to the nucleus (Park et al., 2005). PARP-1 activation-induced cell death is a key mechanism associated with cell death mediated by AIF, with AIF being central to this cytotoxic death pathway (Yu et al., 2002). Activation of PARP-1 contributes to the loss of mitochondrial membrane potential, nuclear translocation of AIF, nuclear agglutination, and, ultimately, cell death. The translocation of AIF occurs after the activation of PARP-1 but before the release of cytochrome C from the mitochondria or the activation of caspase activity such that AIF-neutralizing antibodies can partially disrupt the PARP-1-mediated induction of cell death (Hong et al., 2004). In severe, extensive DNA damage, PARP-1 overactivation can consume available NAD and ATP energy reserves, thus spurring AIF nuclear translocation and facilitating non-caspase-dependent apoptosis (Koh et al., 2005).

Exposure to ionizing radiation increases PARP-1 activity levels significantly within one minute. Inhibiting PARP-1 activity can significantly slow the rate of DNA damage repair (Ryabokon et al., 2008; Ryabokon et al., 2009). PARP-1 inhibitors can increase the degree of single-stranded break (SSB) formation in the DNA, unlike DNA-PK inhibitors, which only affect DSB repair (Zou et al., 2015). Given PARP-1’s critical role in DNA damage repair, using PARP-1 inhibitors to enhance the efficacy of anti-cancer treatments could increase tumor cell sensitivity to radiotherapy or chemotherapy (Underhill et al., 2011). PARP-1 inhibitors have been shown to interfere with PARP-1-mediated SSB/DSB repair. This inhibition of PARP-1 activity can induce apoptosis after exposure to ionizing radiation due to the lack of DSB repair while also promoting necrotic death due to energy deficiencies resulting from reduced PAR production after DNA damage, thus depleting the NAD reservoir (Barth et al., 2006). In most therapeutic settings, PARP-1 inhibitors are combined with chemo- or radiotherapy (Haince et al., 2005; De Soto and Deng., 2006). More detailed reference examples are in the “1. PARP inhibitors” section. PARP-1 activity in active and inactive cells at 24 and 48 h following irradiation was associated with increased ROS production (Chiu et al., 2021), and the inhibition of PARP-1-related ROS production may provide an additional opportunity to increase tumor cell sensitivity to other therapeutic agents (Doroshow, 2006).

## 2 PARP inhibitors

PARP inhibitors disrupt DNA repair, providing ample opportunities for radiosensitization, adjuvant treatment, or chemical synergism. Many such inhibitors have been evaluated in various preclinical and clinical studies, demonstrating their primary function of interfering with PARP-1 activities in DNA repair and transcriptional regulation. The PARP substrate 3-aminobenzamide (3-AB) is a first-generation PARP inhibitor lacking in specificity, although it can inhibit a reported 96% of PARP-1 activity in cells, adversely affecting DNA synthesis, glucose metabolism, and overall cellular viability (Rajawat et al., 2017). 3-AB can promote apoptotic death in HeLa cells by interfering with PARP expression and blocking G2 phase arrest following X-ray irradiation (Masutani et al., 1995). Pairing the PARP inhibitor Veliparib with ionizing radiation can swiftly trigger senescence in tumor cells (Zhang et al., 2016). The PARP-1 inhibitor amplifies ionizing radiation-induced cytotoxicity by suppressing NF-κB activation (Veuger et al., 2009). In K562 cells exposed to ionizing radiation, PARP-1 inhibition can promote ROS production while suppressing PAR production and dramatically reducing the rate of DNA-break repair (Cieślar-Pobuda et al., 2012). Ionizing radiation-induced cytotoxicity sensitivity was observed in both PLC-PRF-5 and HepG2 cell lines one-hour post-treatment with ABT-888 (Guillot et al., 2014). γ-radiation exposure can trigger an overexpression of nuclear PARP, leading to the depletion of both NAD and ATP stores and, subsequently, the induction of oxidative stress. Studies indicate that 3-AB can facilitate wound healing, typically delayed in mice undergoing whole-body γ-irradiation, by modulating the inflammatory and oxidative responses to such irradiation (El-Hamoly et al., 2015). Functionally, 3-AB disrupts the interaction between NAD and the PARP-1 active site. This obstruction inhibits its activation and DNA binding, impairing its ability to recognize DNA sequence breaks (Garon and Dubinett., 2010). Radiosensitive OPSCC cells positive for HPV infection showed upregulation of proteins involved in the SSB and BER repair processes, including PARP1, XRCC1, and PNKP. Treating HPV-negative OPSCCs with the PARP inhibitor Olaparib enhanced their sensitivity to radiation, emphasizing the potential value of combining PARP inhibition and radiotherapy when treating both types of OPSCC (Nickson et al., 2017). Overexpression of the PARP-1 DNA-binding region significantly reduces DSB repair in irradiated cells, indicating that PARP inhibitors have pronounced radiosensitizing activity. Mechanistically, inhibiting PARP-1 may raise the odds of repair failures due to the loss or rapid-to-slow reconnection transitions from PAR-induced DSB due to the expression of a dominant negative form of PARP-1 (Rudat et al., 2001). SSBs can result in rapid PARP-1 activation, and low-dose radiosensitization can be achieved by treating cells with chemical PARP inhibitors in CHO, V79, and T89G cells in the exponential growth phase. Rapidly dividing cells are most sensitive to PARP inhibition, resulting in sensitivity to radiation doses as low as 0.05–0.3 Gy (Chalmers et al., 2004). All of these tumors exhibited increased radiosensitivity when treated with the PARP-1 inhibitor MK-48287 at 50 mg/kg/d (treatment once per hour) to lower intratumoral PAR levels, providing a valuable tool to increase therapeutic efficacy (Wang et al., 2012).

The use of Olaparib to inhibit BER pathway activity resulted in SSB accumulation due to a lack of genomic repair, resulting in the collapse of the replication fork during the S phase of the cell cycle and ultimately contributing to deleterious DSB formation (Bochum et al., 2018). Niraparib activates the cGAS/STING signaling pathway and can thus alter immune responses, and this activity can be further enhanced when combined with PD-L1 blockade, providing a valuable opportunity for clinical intervention (Meng et al., 2021). Rucaparib, a small molecule PARP inhibitor, has been administered to patients with specific resistant and advanced stages of ovarian carcinoma. This treatment resulted in moderate and transient elevations in serum aminotransferase activity in the treated patients. Nevertheless, these changes have not been associated with clinically significant hepatic injury (LiverTox, 2012). The oral PARP inhibitor, Talazoparib, has been used to treat certain breast cancer patients, but the moderate increases in serum aminotransferase levels observed in some cases suggest the possibility of clinically detectable liver damage (Guney, 2019). In clinical trials, Veliparib has demonstrated promising efficacy in treating breast cancers that harbor BRCA mutations and HR pathway deficiencies (HRD). However, additional work is needed to fully explore its optimal dosing and compatibility with other antineoplastic agents (George et al., 2022). In a randomized phase II study of triple-negative breast cancer patients, the PARP inhibitor Iniparib reportedly exhibited promising activity (Mateo et al., 2013). The selective and potent PARP-1 inhibitor AZD5305 also has PARP-1-DNA trapper activity, shown in a preclinical *in vivo* patient-derived xenograft (PDX) model of BRCA mutant breast cancer (HBCx-17) (Johannes et al., 2021). The investigational PARP-1 and PARP-2 inhibitor Pamiparib has also shown promising antitumor efficacy, suppressing the proliferation of tumor cell lines harboring BRCA1/2 mutations or HRD (Wang et al., 2020). When treating platinum-sensitive relapse ovarian cancer cells with germline BRCA1/2 mutations, Fluzoparib also exhibited good safety and antitumor activity profiles (Li et al., 2021). BGP-15 reportedly reduced the ischemia-reperfusion-induced self-ADP-ribosylation of nuclear PARP and the mono-ADP-ribosylation of GRP78, an ER chaperone (Szabados et al., 2000). E7449 can inhibit the enzymatic activity of PARP, trapping PARP-1 in association with damaged DNA in a manner that contributes to higher rates of cytotoxic cell death. Consistently, cells with defects in DNA repair pathways showed high levels of E7449 sensitivity, which exhibited single-agent activity in BRCA-derived xenograft models, had its activity enhanced in combination with chemotherapeutic agents, and suppressed Wnt/β-catenin pathway signaling in colon cancer cells, likely via inhibition of TNKS (McGonigle et al., 2015). BYK204165 is an isoquinolindione that reportedly exhibits 100-fold greater selectivity for PARP-1, making it a promising tool for studies specifically focused on the effects of inhibiting PARP-1 (Eltze et al., 2008). The PARP inhibitor A-966492 can increase the radiosensitivity of U87MG spheroids, while TPT increases such radiosensitivity by interfering with DNA damage repair and resulting in S-phase cell accumulation. Combining the topoisomerase I inhibitor TPT and A-966492 can enhance tumor cell radiosensitivity in an additive manner (Koosha et al., 2017). DPQ and PJ-34 can suppress inflammation and other deleterious effects associated with the overexpression of PARP-1 while increasing cell viability, underscoring their potential clinical utility (Scalia et al., 2013). It has been suggested that 3-AB can significantly augment acetylcholine-induced, endothelium-dependent, nitric oxide-mediated vasorelaxation, normalizing the function of endothelial tissue following exposure to hydrogen peroxide (400 μM) (Radovits et al., 2007). Details of the mechanisms of the inhibitors are listed in Table 1.

**TABLE 1:** PARP-1 inhibitors.

Name	Mechanism	References
Olaparib	Olaparib (AZD2281) inhibits PARP-1 and PARP-2 at single-digit nanomolar concentrations. Olaparib reportedly exhibits single-agent efficacy against breast cancer cells harboring BRCA1 deficiencies, has been applied to SW620 cell lysates, and can reportedly inhibit PARP-1 with an IC50 of ∼6 nM, fully inhibiting this enzyme at concentrations of 30–100 nM	Menear et al. (2008)
Niraparib	Niraparib can suppress DNA damage repair, exerting antitumor activity by promoting apoptotic cell death	Jones et al. (2009), Wang et al. (2012), Bridges et al. (2014)
Rucaparib	Rucaparib can inhibit NF-κB activation in response to DNA damage independent of SSB repair, increasing tumor cell sensitivity to irradiation. Rucaparib can also avoid the toxic effects associated with many classical NF-κB inhibitors without disrupting other key inflammatory activities. At a concentration of 1 μM, Rucaparid can reportedly inhibit 97.1% of PARP-1 activity in permeabilized D283Med cells	Hunter et al. (2012), Shirley (2019)
Talazoparib	The highly potent and orally active PARP-1/2 inhibitor Talazoparib (BMN-673) with anticancer activity exhibits respective Ki values of 1.2 nM and 0.87 nM for PARP-1 and PARP-2	Wang et al. (2016)
Veliparib	Veliparib, also known as ABT-888, has been tested and shown to be inactive against another enzyme that relies on NAD + for its catalytic activity (SIRT2, >5,000 nM)	Donawho et al. (2007)
Iniparib	Iniparib can modify cysteine-containing proteins within tumor cells in a nonspecific manner, and can weakly suppress SSB repair at a dose of 100 μM, with this inhibition being reversed by PARP-1 knockdown	Liu et al. (2012), Yin et al. (2017)
PJ34	PJ34 can inhibit the enzymatic activity of PARP (IC50: 110 ± 1.9 nM). Its neuroprotective properties were assessed via LDH assay in PC12 cells wherein it was shown to suppress cell death in a dose-dependent manner at doses from 10^–7^ to 10^–5^ M	Iwashita et al. (2004)
AZD5305	The potent, orally active PARP inhibitor AZD5305 has exhibited efficacy in PDX and xenograft model systems	Johannes et al. (2021)
Pamiparib	Tumor cells harboring defects in the homologous recombination pathway exhibit high levels of Pamiparib sensitivity, with this inhibitor exhibiting high levels of *in vitro* and *in vivo* activity in tumors harboring BRCA mutations	Friedlander et al. (2019)
Fluzoparib	The orally active PARP-1 inhibitor Fluzoparib (SHR3162) exhibits an IC50 of 1.46 ± 0.72 nM in cell-free assays, and exhibits promising antitumor activity due to its ability to selectively impair the proliferation of cells exhibiting HR deficiencies, sensitizing HR-deficient and HR-proficient cells to exposure to other cytotoxic compounds or treatments. *In vivo* work suggests that Fluzoparib exhibits promising pharmacokinetic characteristics and that it is applicable for use in research focused on BRCA1/2-mutant relapsed ovarian cancer	Wang et al. (2019)
3-Aminobenzamide	3-Aminobenzamide can inhibit over 95% of PARP activity (>1 μM) without inducing any significant toxicity in treated cells. INO-1001 can sensitize CHO cells to radiation by interfering with DNA repair following radiation exposure	Brock et al. (2004)
AZD-2461	AZD-2461 can facilitate the resistance of BRCA-2-deficient murine breast cancer cells (KB2P3.4) and high levels of P-gp expression	O'Connor et al. (2016)
BGP-15	AZD-2461 can inhibit PARP-1, PARP-2, and PARP-3 with respective IC50 values of 5 nM, 2 nM, and 200 nM. It can also inhibit SSB repair in human A549 cells at a dose of 500 nM, and can cause resistance and expression of high levels of P-gp in BRCA2-deficient KB2P3.4 murine breast cancer cells. It also exhibits cytotoxicity when used to treat BT-20 cells at concentrations of 5–50 μM, increasing the frequency of these cells in the S and G2 phases of the cell cycle while having a limited effect on SKBr-3 cell cycle progression at doses of 5–20 μM	O'Connor et al. (2016), Węsierska-Gądek et al. (2015)
NMS-P118	NMS-P118 is a metabolically stable PARP-1/2 inhibitor that exhibits lower levels of *in vitro* myelotoxicity than does Olaparib. NMS-P118 can moderately inhibit two of eight tested cytochrome P450 family members (CYP-2B6 IC50: 8.15 μM; CYP-2D6 IC50: 9.51 μM), impairing bone marrow cell proliferation at levels 5- to over 60-fold lower than Olaparib in different species	Papeo et al. (2015)
AG14361	AG14361 is capable of suppressing the growth of breast cancer cells, exhibiting respective IC50 values of 17 μM and 25 μM for 92 J-wt-BRCA1 and 92 J-sh-BRCA1 cells. It can also promote abnormalities in the cell cycle while driving the activation of caspase-3/7 and suppressing NF-κB signaling[25]. AG14361 (0.4 μM) can enhance the ability of compounds that inhibit topoisomerase I to suppress cell growth and survival without adversely affecting cleavable complex formation or reversal, increasing camptothecin-induced DNA SSB persistence	Smith et al. (2005), Vazquez-Ortiz et al. (2014)
Venadaparib	Venadaparib can interfere with SSB repair and has been studied for the treatment of solid tumors	Myongjae et al. (2018), Yong et al. (2021)
E7449	E7449 does not inhibit PARP3 or PARP6-16, but can trap PARP-1 in association with damaged DNA, influencing DNA repair pathway activity. It can readily suppress cells exhibiting HR pathway deficiencies y (BRCA1 and 2, CtIP, Rad54), in addition to suppressing Wnt signaling in SW480 cells at a dose of 10 μM	McGonigle et al. (2015)
BYK204165	BYK204165 can inhibit human PARP-1 enzyme activity with a pKi of 7.05 in kinetic experiments, and is a low-potency PARP inhibitor in C4I cells (pIC50: 5.75)	Eltze et al. (2008)
A-966492	A-966492 is a potent PARP inhibitor that potently inhibits PARP-1 (Ki = 1 nM) with an EC50 of 1 nM in whole-cell assays	Penning et al. (2010)
Nudifloramide	Nudifloramide (2PY) is an NAD degradation end product that can inhibit the activity of PARP-1 *in vitro*	Rutkowski et al. (2003)
EB-47	The selective PARP-1 inhibitor EB-47 mimics the NAD + substrate, extending from the nicotinamide to the adenosine subsite, providing an opportunity to improve radiotherapy and chemotherapy outcomes	Haikarainen et al. (2013)
WD 2000–012547	The PARP-1 inhibitor WD 2000–012547 (Compound 66) exhibits a pKi of 8.221	Fatima et al. (2014)
DPQ	The PARP-1 inhibitor DPQ can suppress NMDA-induced activation of PARP, protecting cells against ATP depletion and alleviating neuronal injury in a model of excessive NMDA exposure	Meli et al. (2004)
E7016	E7016 is capable of inhibiting DNA repair, increasing tumor cell sensitivity to radiation exposure *in vitro* and *in vivo*	Russo et al. (2009), Lai et al. (2011)
GPI 15427	GPI 15427 can enhance the antitumor efficacy of temozolomide when used to treat hematological or solid tumors, and can cross the blood-brain barrier	Tentori et al. (2003), Tentori et al. (2005)
NMS-P515	NMS-P515 can effectively inhibit PARP-1 in biochemical and cellular assays, with respective Kd and IC50 values of 0.016 μM and 0.027 μM. The stereospecific inhibitory activity of NMS-P515 has been explored through cocrystal structure analyses	Papeo et al. (2019)
7-Methylguanine. (7-MG)	7-MG can compete with NAD for binding to the active site of PARP-1, interacting through both hydrogen binding and nonpolar interactions with Gly863, Ala898, Ser904, and Tyr907 residues. By promoting the formation of a PARP-1 nucleosome complex, 7-MG can suppress PARP-1 automodification that is DNA-dependent	Nilov et al. (2020)
7-azaindole-1-carboxamide	This compound readily binds to G-quadruplex structures, including the human telomere repeat sequence, d(TTAGGGT) 4, and the Pu22 c-MYC promoter sequence. G-quadruplex model formation suggests that it may function by stabilizing these structures to inhibit PARP activity	Dallavalle et al. (2021)
Nicotinamide	Nicotinamide, also known as vitamin B3, is an essential cellular precursor for NAD production that is related to the development, death, and survival of neurons	Salech et al. (2020)
Crellastatin A	PARP-1 has been identified as the main cellular target of crellastatin A through *in silico* and biochemical research, functioning as a PARP-1 inhibitor	Morretta et al. (2020)
AG-690/11026014	AG-690 may be able to inhibit PARP-1 and to thereby reverse NAD depletion, restoring SIRT6 activity and mediating anti-hypertrophic efficacy. This compound can also suppress NADPH oxidase 2/4 activity, reducing ROS generation and potentially thereby contributing to PARP-1 inhibition	Liu et al. (2014)
HYDAMTIQ	HYDAMTIQ can lower TGF-β expression levels and suppress TGF-β/SMAD signaling pathway activity	Lucarini et al. (2017)
5-Aminoisoquinolinone	The 5-AIQ treatment of BTBR mice was associated with increased Helios, FOXP3, and IL-10 expression as well as reductions in the mRNA levels of IL-9, IL-17A, and GATA3	Alhosaini et al. (2021)
BTH-8	BTH-8 can induce DNA DSB formation, resulting in γ-H2AX foci accumulation and inhibiting PAR formation. In HCC-1937 cells, BTH-8 was reported to induce pronounced G2/M cell cycle arrest and apoptotic death	Guo et al. (2020)
NU1025	p53 activity can influence glioblastoma multiforme cell sensitivity to combination treatment with PARP inhibitors and ionizing radiation	Sabbatino et al. (2014)
2″-hydroxygenkwanol A	This compound is capable of efficiently interacting with the PARP catalytic domain in the NAD binding pocket.	Dal Piaz et al. (2015)
4-AN	4-AN can contribute to the chemosensitization of HepG2 cells by suppressing PARP-1 and thereby interfering with DNA damage repair and abrogating the G2/M cell cycle checkpoint, resulting in ATO sensitization	Luo et al. (2015)
MP-124	MP-124 is a PARP-1 inhibitor that reportedly competes with the binding of NAD as a PARP substrate. It is actively undergoing exploration as a potential neuroprotective drug to protect against acute ischemic stroke	Yamamura et al. (2015)
SIR	SIR can lower glutathione levels while increasing levels of disulfide glutathione, suppressing glutathione reductase activity	Jacewicz et al. (2009)
INO-1001	INO-1001 has been shown to be safe in the context of DNA repair	Hauser et al. (2006)
MC2050	MC2050 can protect cells against hydrogen peroxide-induced damage and can alter target gene expression patterns	Mosca et al. (2011)

The potency of PARP inhibitors was shown to be associated with both the recommended therapeutic dosage and the severity of adverse effects. In 2018, the FDA authorized Talazoparib to treat HER2-negative and BRCA-mutated breast cancer. Cases with breast cancer who have the BRCA mutation had a 21%–37% survival rate when given 1 mg/day of Talazoparib (Turner et al., 2019). Cases with advanced breast cancer with BRCA mutations who received Talazoparib had a higher median survival rate (62.6% vs. 27.2%) than those who received conventional treatment (Litton et al., 2018). Talazoparib, the most potent PARP inhibitor, has been associated with the highest incidence of anemia. The lowest effective dosage is 1 mg orally once daily (de Bono et al., 2017; Litton et al., 2018; Pilie et al., 2019). Talazoparib has been shown to enhance the efficacy of *in vivo* radiotherapy and chemotherapy in various cancers, including lung cancer, colorectal cancer, glioblastoma, and serous ovarian cancer xenotransplantation (Calabrese et al., 2004; Donawho et al., 2007; Bi et al., 2018). Specifically for glioblastoma, a study found that Talazoparib has a greater radiosensitization effect on stem cells compared to other PARP inhibitors (including Olaparib and AG14361), even at lower concentrations (Lesueur et al., 2018). Talazoparib has been shown to enhance the efficacy of *in vivo* radiotherapy and chemotherapy in various cancers, including lung cancer, colorectal cancer, glioblastoma, and serous ovarian cancer xenotransplantation (Calabrese et al., 2004; Donawho et al., 2007; Bi et al., 2018). Specifically for glioblastoma, a study found that Talazoparib has a greater radiosensitization effect on stem cells compared to other PARP inhibitors (including Olaparib and AG14361), even at lower concentrations (Lesueur et al., 2018). The European Medicines Agency (EMA) authorized Olaparib in 2014 as a maintenance therapy for BRCA mutant ovarian malignancies when platinum chemotherapy can be tolerated following three or more lines of chemotherapy. Currently, the standard dosage is 300 mg twice/day. Patients with advanced BRCA1/2-mutated ovarian cancer who have progressed or relapsed following platinum-based chemotherapy do not benefit more from treatment with Olaparib than those who get pegylated liposome doxorubicin (PLD) (Kaye et al., 2012). The use of Olaparib as a radiation sensitizer during breast radiation therapy may not be associated with an increased risk of late complications. Therefore, we suggest that in future clinical trials, when Olaparib is combined with breast radiation therapy, the radiation sensitization dose should be 200 mg twice a day to evaluate the antitumor effect of the combination (Loap et al., 2022). In 2016, the FDA authorized Rucaparib to treat advanced ovarian cancer cases with somatic BRCA1/2 or germline mutations. The median survival was extended from roughly 5 months with a placebo to 16.6 or 21 months with Niraparib (300 mg once/day) and Rucaparib (600 mg twice/day), according to phase 3 studies (Coleman et al., 2017; Mirza et al., 2016). Single-agent administration of Veliparib did not improve the clinical outcomes for patients with pancreatic and ovarian cancers (Coleman et al., 2015; Lowery et al., 2018). Nevertheless, combining Veliparib with Paclitaxel and Carboplatin enhanced survival after maintenance treatment more than Paclitaxel and Carboplatin alone in a recent phase 3 study of advanced serous ovarian malignancy (Coleman et al., 2019). Veliparib stands out as the most promising PARP inhibitor with therapeutic implications, capable of being administered alongside standard chemotherapy without eliciting undesirable toxicity. A Phase I multicenter study was initiated with 30 participants diagnosed with either inflammatory or recurrent breast cancer localized to the surgical resection area. This trial involved the administration of Veliparib concomitant with radiotherapy targeting the chest wall and regional lymph nodes. Despite the highest experimental dose, severe acute toxicity did not surpass 30%. However, nearly half of the surviving patients exhibited grade 3 adverse events after 3 years, underscoring the critical necessity of long-term toxicity monitoring in trials involving radiation sensitizers (Jagsi et al., 2018).

## 3 The role of PARP-1 in DSB and SSB repair

PARP-1, the most abundantly expressed member of the PARP family, acts as a key component in the BER pathway (Anwar et al., 2015). In addition to its direct effects as a mediator of DNA repair, PARP-1 is also associated with inflammation, post-transcriptional gene regulation, and cell death (Langelier et al., 2008; Tao et al., 2008; Rajawat et al., 2017). PARP-1, when drawn to sites of DNA damage, plays an instrumental role in maintaining or regaining genomic stability, thereby aiding in cell survival (Veuger et al., 2003; Chalmers et al., 2004; Haince et al., 2008). Upon the incidence of DNA damage, PARP-1 is activated, catalyzing the splitting of NAD + into nicotinamide and ADP-ribose. This reaction leads to the formation of elongated branches of ADP-ribose polymers capable of modifying target proteins (Aguilar-Quesada et al., 2007). Overactivation of PARP, however, may result in NAD+ and ATP depletion, compromising the energy supply at a cellular level and potentially provoking necrotic cell death (Sodhi et al., 2010; Underhill et al., 2011).

High glucose levels have been associated with the activation of PARP1 in endothelial cells (Garcia Soriano et al., 2001; Du et al., 2003; Piconi et al., 2004). This activation is partly due to DNA damage and oxidative stress induction. Thus, PARP-1 expression may be associated with ROS production in cells. If the resultant oxidative stress is not adequately controlled, it can cause DNA breaks that contribute to PARP-1 overactivation and potential necrotic or apoptotic cell death (Zakaria et al., 2016). Inhibition of PARP can protect against ROS-mediated cell death and preserve the mitochondrial membrane potential in an MKP-1 and ATF4-dependent manner (Hocsak et al., 2017). Recent work has demonstrated that PARP-1 activation is directly triggered by ROS-induced damage to the DNA, ultimately contributing to a loss of cellular viability (Cole and Perez-Polo., 2002). Although research has frequently explored PARP-1 regulation of DNA in tumor cells, less is known about PARP-1-mediated regulation in normal cells.

Tumor cell resistance to radiotherapy is primarily mediated by robust DNA repair functionality. Therefore, actions such as silencing, inhibiting, or knocking out PARP-1 can significantly enhance the efficacy of radiotherapy by impairing DNA repair. PARP-1 is an important SSB and DSB repair mediator, orchestrating appropriate cellular responses after radiation exposure. (Veuger et al., 2003; Chalmers et al., 2004; Chen et al., 2018; Sefer et al., 2022). By targeting these DNA repair mechanisms, PARP inhibitors can increase the cytotoxic effects of radiotherapy and chemotherapy (Curtin, 2012; Steffen et al., 2013). Exposure to ionizing radiation can induce both SSB and DSB in DNA, which can be repaired by activating appropriate DNA repair enzymes, thus preserving cell and tissue function. Several clinical trials have validated the potential of PARP inhibitors for use as a radiosensitizing treatment, while experimental studies indicate an increase in radiosensitivity following the silencing of PARP-1. This enhancement in radiosensitivity is somewhat distinct from the effects observed under PARP-1 inhibition, which predominantly impacts cells in the S-phase (Godon et al., 2008). Olaparib, which inhibits PARP-1/2/3, modifies SSB repair and displays significant radiosensitizing effectiveness when administered for treating Lewis lung cancer cells and tumor xenografts. This effectiveness might be associated with an elevation in DNA DSB formation and an upregulation of the pro-apoptotic Bax/Bcl-2 upon irradiation (Wang et al., 2016). Observations of increased PARP-1 expression in response to adaptive radiation imply a potential role for this enzyme in cellular adaptation to exposure to low-dose radiation (Cheng et al., 2010). This radiation-induced PARP-1 activation can also drive the migratory and invasive activity of CNE-2 nasopharyngeal carcinoma cells (Lu et al., 2018). One study found that high linear energy transfer (LET) ionizing radiation was only sufficient to inhibit the Ku (XRCC5)-dependent NHEJ pathway but not the PARP-1-dependent NHEJ or homologous recombination repair (HRR) pathways. Exposure to high LET, facilitated by highly charged particles, can lead to impaired DNA repair and more DSB fragments that are less than 40 base pairs in size. This prevents the Ku protein from binding to the ends of these DSB fragments more effectively than exposure to a dose of low LET ionizing radiation from X-rays, which delays Ku-dependent repair (Zhang, 2008). PARP can be activated in a dose-dependent manner in response to synchrotron radiation X (SRX)-associated tissue damage. At the same time, this is inhibited by treatment with the antioxidant n-acetylcysteine (NAC), highlighting the role of oxidative stress in the mediation of SRX-induced PARP activation (Ying, 2013).

PARP-1 plays diverse and complex roles in repairing DNA damage and preserving genomic integrity (Martin-Hernandez et al., 2017; Ray Chaudhuri and Nussenzweig., 2017). In the context of DNA repair, the three main steps are detecting DNA damage, the PAR-mediated recruitment of repair-related factors, and the PAR-mediated regulation of key biochemical processes. A multitude of studies elaborates on the involvement of PARP-1 in the SSB repair and BER pathways (Masson et al., 1998; Trucco et al., 1998; Dantzer et al., 2000; Lavrik et al., 2001; Prasad et al., 2001; El-Khamisy et al., 2003; Leppard et al., 2003; Fisher et al., 2007), with SSB damage detection being followed by the PAR-mediated recruitment of XRCC1 to these sites of DNA damage.

**FIGURE 3:**
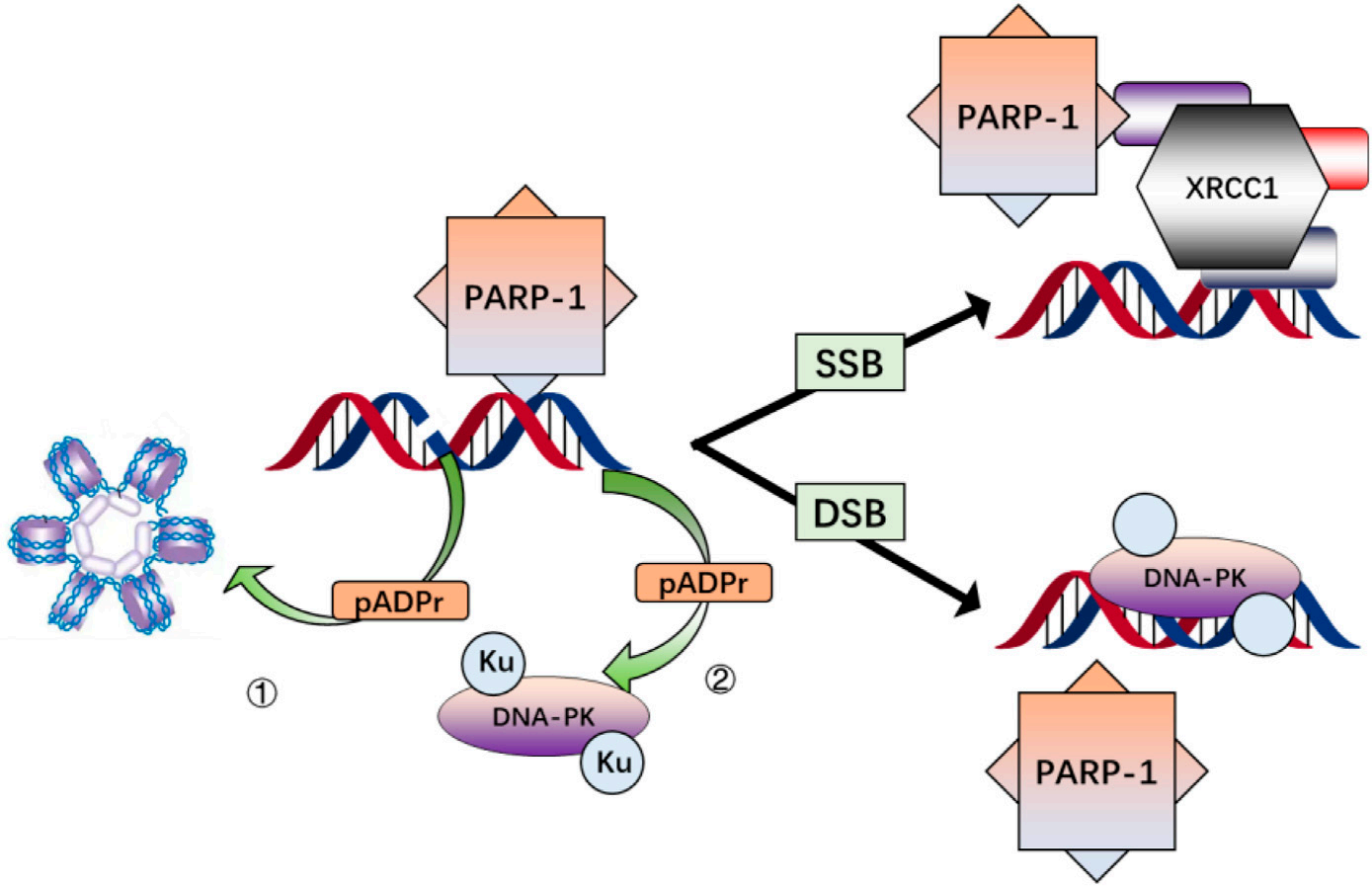
Simplified schema illustrating the major interactions of PARP-1 after DNA damage. PARP-1 is activated by binding to single-or double-strand breaks in DNA, resulting in the modification of nuclear proteins via poly(ADP-ribosylation) (pADPr) and direct interaction while histone modification contributes to chromatin relaxation 1); pADPr of DNA-PKcs and physical associations with Ku proteins enhance DNA-PK recruitment and activation 2); pADPr of components of the base excision repair pathway and direct interaction of PARP-1 with XRCC1 facilitates assembly and activation of the base excision repair complex.

PARP-1 is a highly conserved nuclear protein that binds rapidly to SSB and DSB sites in the genome and exhibits PAR catalytic activity (Herceg and Wang., 2001). The binding of PARP-1 to DNA damage sites and its PAR catalytic activity triggers its enzymatic activation. This activation leads to the modification of various nuclear proteins *via* the covalent attachment of branching PAR chains (Figure 3) (D'Amours et al., 1999). Cells and animals deficient in PARP-1 present marked genomic instability and diminished BER activity, highlighting this enzyme’s critical role in cellular responses to low doses of ionizing radiation (Shall and de Murcia., 2000).

## 4 Interactions between PARP-1 and p53/ROS

ROS and p53 mutually regulate one another in a feedback cycle (Maillet and Pervaiz, 2012), with p53 serving to protect the genome from ROS-induced DNA damage. At baseline conditions, minimal p53 expression is necessary to stimulate the expression of antioxidant genes sufficient for mitigating oxidative stress (Sablina et al., 2005). In addition to regulating DNA damage, apoptotic death, and senescence, p53 can modulate autophagy. Consequently, depletion or inhibition of p53 can promote autophagy in mice, humans, and nematode cells (Lakin and Jackson., 1999; Tavernarakis et al., 2008). PARP does not subject DNA ligase I to PAR modification but regulates DNA repair by altering chromosomal structures to ensure that the damaged sites are accessible to the appropriate repair enzymes (Bhat et al., 2006). Evidence shows that p53 can modulate glioblastoma cell sensitivity to combined TPT, radiotherapy, and PARP inhibitor treatment. Specifically, cells with activated p53 have greater sensitivity to a combination of radiotherapy and PARP inhibitor treatment, while cells with inactive p53 are more sensitive to combinations of TPT and PARP inhibitors (Sabbatino et al., 2014). The combination of radiation and emodin can induce PARP-1 cleavage and the downregulation of epigenetic signaling mediators, including JMJD1A and JMJD2B, thus contributing to apoptotic death. Accordingly, emodin overcame HepG2 cell radioresistance by promoting the upregulation of apoptotic signaling molecules and downregulation of pro-proliferation signaling factors (Hwang et al., 2015). Trans-dominant inhibition of PARP-1, resulting from the overexpression of the PARP-1 DNA-binding domain in MCF-7 cells, can lead to a reduction in p21 induction and G1 cell cycle arrest instigated by p53 following exposure to ionizing radiation. Inhibiting the functionality of endogenous PARP-1 can thus suppress p53 transactivating activity following radiation exposure. PARP-1 is thus a key regulator of p53-mediated responses to DNA damage (Wieler et al., 2003). The p53 status of BRCA1 and HR-proficient tumors can determine the degree to which PARP inhibitor treatment sensitizes them to ionizing radiation, providing an opportunity to identify patients with the greatest chance of benefiting from the combination treatment (Sizemore et al., 2018). Indeed, capitalizing on the mutational status of p53 in human tumors, in conjunction with the inactivation of PARP-1, could potentially render otherwise resistant cells susceptible to radiotherapy.

Various strategies have been used to inhibit PAR synthesis. Studies in cells lacking PARP activity have emphasized the critical role of poly(ADP-ribosyl)ation in regulating DNA repair and DNA metabolism (D'Amours et al., 1999). Investigations using PARP knockout mice revealed beneficial roles of PARP in maintaining genomic integrity and survival after exposure to whole-body γ-radiation. The capacity of PARP to hinder recombination at sites of DNA strand breaks was clearly shown in studies involving PARP and another key DNA strand break-binding protein, the DNA-dependent protein kinase (D'Amours et al., 1999; Le Rhun et al., 1998). Decreases in the covalent PAR modification of p53 also coincided with increases in the expression levels of p53-responsive genes, including Fas and Bax, indicating a possible role for poly-(ADP-ribosyl)ation as a regulator of the function of p53 while highlighting the importance of PARP and PAR modification during the early stages of apoptotic cell death (Prives and Hall., 1999; Simbulan-Rosenthal et al., 1999). The activation of p53 and its upregulation occurs within minutes to hours following DNA damage (Prives and Hall., 1999), while PARP-1 can respond to DNA damage in seconds to minutes (D'Amours et al., 1999), indicating a potential upstream role for poly(ADP-ribosylation) in the regulation of the expression and function of p53 following genotoxic stress exposure.

**FIGURE 4:**
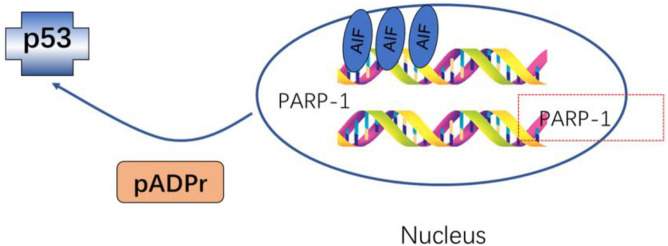
pADPr of p53 and transcription factors influence cell cycle progression and reduce overall levels of gene transcription.

The simultaneous mutation of both PARP-1 and Wrn results in a more pronounced shift in gene expression profiles than those observed when either gene is mutated in isolation. Moreover, cultured cells with mutations in both genes exhibit extensive dysregulation of signal transduction, cell cycle progression, metabolism, embryonic development, and apoptosis-related genes. *In vivo* studies using dual-gene mutant mice have demonstrated higher rates of embryonic developmental defects, apoptosis, and elevated levels of oxidative damage and intracellular phosphorylation in adult tissue samples (Deschênes et al., 2005) (Figure 4).

**FIGURE 5:**
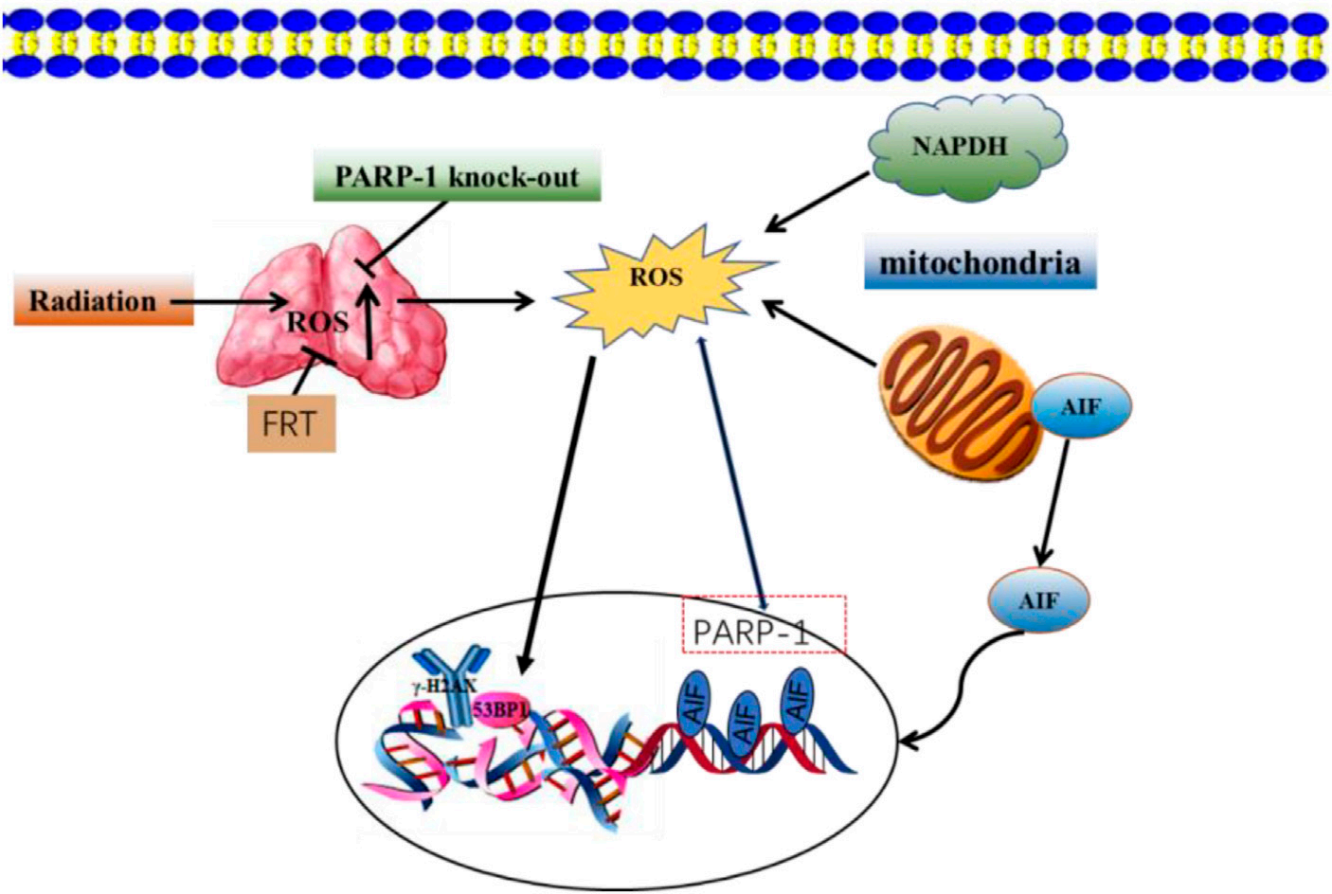
FRT protects against radiation damage by regulating the PARP-1/ROS/DNA pathway and by inhibiting the activation of PARP-1/AIF.

Oncogenesis is a complex process driven by exogenous and endogenous stimuli, including endogenous ROS-mediated damage to the deoxyribosyl DNA backbone (Valko et al., 2004; Guo et al., 2016. The p53 protein is capable of regulating ROS-related stress and genomic damage. Correspondingly, the loss of p53 function enhances the radiosensitizing activity of Olaparib in both isogenic and non-isogenic cell line models. This coincides with increased levels of PARP-1 expression in bladder cancer cell lines and associated tumors. The simultaneous loss of p53 and impairment of ataxia-telangiectasia mutated (ATM) activity can produce more robust radiosensitivity (Liu et al., 2018). Investigations into the impact of PARP-1 inhibition on ROS generation and DNA repair post-exposure to ionizing radiation revealed no significant variances in DNA strand breaks between cells treated with PARP inhibitors and those that were untreated after a period of 3 h. However, at 12 and 48 h following irradiation, cells treated with PARP inhibitors displayed markedly higher surges in ROS levels compared to their untreated counterparts (Cieślar-Pobuda et al., 2012). Interfering with PARP-1 activity can also increase ROS levels in ovarian cancer cells, leading to DSB in the DNA. NAC is an antioxidant that inhibits PARP-1-mediated dysregulation of cell proliferation, highlighting a protective role for PARP-1 in augmenting the antioxidant capacity of ovarian cancer cells (Hou, 2015). A combined treatment approach using radiation and flavonoids from *Rosa roxburghii* Tratt (FRT) can enhance radioresistance. This improvement is mediated by PARP-1-dependent cell death pathways, which involve PARP-1 activation and ROS generation inhibition. The combination of FRT treatment and PARP-1 knockout significantly reduces ROS levels in cells and prevents the mitochondrial translocation of AIF (Figure 5) (Xu et al., 2020).

## 5 Interactions between PARP-1 and NF-κB/DNA-PK

### 5.1 Interactions between PARP-1 and DNA-PK

*In vitro* and *in vivo* analyses have demonstrated that the loss or inhibition of PARP-1 function can compromise DNA repair, making tumors more sensitive to chemotherapy and radiotherapy (Huang and Huang, 2006). In nasopharyngeal carcinoma cells, PARP-1 can activate autophagy through the AMPK/mTOR pathway such that interfering with PARP-1 or AMPK activity can suppress autophagic activity and increase CNE-2 nasopharyngeal carcinoma cell sensitivity to radiation treatment (Chen et al., 2015; Chen, 2016). DSBs that develop in the genome following radiation exposure are generally repaired by the NHEJ pathway, which relies on DNA-PK activity. PARP-1, while best known for its role in SSB repair, can also mediate DSB repair. DNA-PK and PARP-1 can promote the rapid repair of DSBs caused by ionizing radiation, and inactivating these enzymes does not yield additive effects, indicating that they cooperate to promote the same DSB repair response pathway (Mitchell et al., 2009). DNA-PK and PARP-1 are also involved in rapid changes in radiosensitivity in cells exposed to short pulses of ionizing radiation. The inactivation of DNA-PK through Wortmannin can intensify the cytotoxicity induced by irradiation without modifying the oscillatory character of early radiation responses. On the contrary, 3-AB mediated inhibition of PARP disrupts this oscillatory response, a disruption also observed in 3T3 fibroblasts with silenced PARP-1 (Fernet et al., 2000). The function of nuclear receptor subfamily 4 group A (NR4A) proteins is to directly target DNA-PK catalytic subunit (DNA-PKcs) that have been poly-ADP-ribosylated, facilitating its autophosphorylation, which promotes DNA-PK kinase assembly at DNA damage sites. Selective targeting of the PAR-binding pocket of NR4A presents an opportunity for cancer therapy (Munnur et al., 2019). Replication forks may be better protected, repaired, and restarted if XRCC1 is recruited in a PARP and DNA-PK-dependent approach (Ying et al., 2016). Using ATM-deficient cells, it was found that the combined use of AZD7648, a DNA-PK inhibitor, and Olaparib, a PARP inhibitor, increased genomic instability in cells lacking ATM, leading to reduced cell growth and the promotion of apoptosis (Fok et al., 2019). Treatment with PARP inhibitors increased the phosphorylation of DNA-PK substrates and stimulated NHEJ in cells lacking HR, leading to potential errors. Notably, inhibition of DNA-PK activity could reverse the genomic instability induced by inhibiting PARP (Patel et al., 2011). The PARP inhibitor Olaparib was observed to induce both phosphorylation and stabilization of p53 in a DNA-PK-dependent manner in mantle cell lymphoma cells lacking ATM and promote the expression of p53-responsive factors responsible for regulating cell-cycle checkpoints. At the same time, Olaparib toxicity could be reduced by inhibiting DNA-PK in these cells (Williamson et al., 2012). Animal investigations indicate that using the PARP inhibitor Olaparib and the DNA-PK inhibitor NU7441 in conjunction with ionizing radiation boosts the suppression of HPV-negative head and neck squamous cell cancer (HNSCC), indicating that this may be a useful and novel approach to treating cases with HPV-negative HNSCC (Zeng et al., 2020).

### 5.2 Interactions between PARP-1 and NF-κB

The transcription factor NF-κB is an essential regulator of inflammatory signaling activity and oncogenic processes. Constitutive NF-κB expression is seen in tumors, promoting the upregulation of proliferation, invasion, metastasis, and angiogenesis-related genes. Several tumors exhibit altered or elevated basal NF-κB activity levels that coincide with increased resistance to radiotherapy and chemotherapy (Martin-Oliva et al., 2006; Sorriento et al., 2012). Exposure to ionizing radiation can induce complex, multifaceted responses in mammalian cells. These responses include activating pro-survival pathways that lead to the transient activation of specific transcription factors. These factors include NF-κB and members of the signal transducers and activators of the transcription (STAT) family, which serve as core mediators of pro-inflammatory and oncogenic signaling activity. These factors ultimately drive the expression of a diverse range of anti-apoptotic and pro-inflammatory genes that contribute to enhanced cell survival, invasivity, and angiogenesis, rendering tumors more resistant to radiation exposure. The upregulation of inflammatory cytokines, including IL-1β, IL-6, and TNF-α in response to radiation can also cause symptoms such as fatigue, localized inflammation, and pain in patients undergoing cancer treatment (Deorukhkar and Krishnan, 2010). LRP16 can interact with IκB kinase (IKK) and PARP-1, thereby facilitating the lesion-specific recruitment of these factors and the recruitment of PIASy to IKK following DSB formation (Wu et al., 2015). In the ovaries, irradiation can enhance the expression of PARP-1 and NF-κB, thereby promoting the upregulation of inflammatory factor mRNAs, including IL-6, IL-8, and visfatin, together with reduced IL-10 and increased iNOS and COX-2 protein expression. Resveratrol treatment can reportedly restore function to the ovaries by increasing levels of AMH and reducing inflammatory activity through mechanisms that largely center around PPAR-γ and SIRT1 upregulation and the consequent inhibition of NF-κB-induced inflammatory cytokine signaling (Said et al., 2016). Emerging evidence indicates a role of PARP-1 in the activation of NF-kB. Research has shown that the application of PARP-1 inhibitor AG14361 on breast cancer cell lines, which constitutively express NF-kB, successfully suppressed the expression of a luciferase reporter gene activated by ionizing radiation. This observation was mirrored in mouse embryonic fibroblasts, irrespective of their PARP-1 or NF-kB p65 expression. PARP-1 is thus a key mediator of radiation-driven NF-kB activation, with the potentiation of radiation-mediated cytotoxicity by AG14361 treatment resulting from NF-kB inhibition (Veuger et al., 2009). The shRNA-mediated silencing of EWS-FL1 ablated the impact of inhibiting PARP-1 on Ewing sarcoma cell radiation responsivity. Combining 4 Gy radiation with PARP-1 inhibition reduced the growth of SK-N-MC xenograft tumors, demonstrating that inhibiting PARP-1 in Ewing sarcomas can synergistically prolong and exacerbate radiation-induced DNA damage-promoting apoptotic death in an EWS-FLI1-dependent manner (Lee et al., 2013).

Onjisaponin B (OB) is an inhibitor of NF-κB signaling that can protect against endotoxin-induced liver damage (Fu et al., 2016). OB can also prevent the proliferation of osteoclasts and protect against bone loss by downregulating NF-κB (Yang et al., 2015). OB may also control NF-κB signaling through PI3K/AKT pathways, and this action is independent of radiation. However, the mechanism by which OB confers radioprotection to cells by inhibiting p65 remains unclear. Both caspase-3 and NF-κB become activated following radiation exposure. Notably, in a neonatal retinal hypoxia model, NF-κB is reported to promote caspase-3 activation, which contributes to retinal ganglion cell death.

Inhibitors of NF-κB can also suppress caspase-3-dependent apoptotic cell death (Li et al., 2020). Activating NF-κB leads to additional caspase-3 activation, while suppressing NF-κB activity suppresses the activity of caspase-3 (Cao et al., 2013). Given the ability of NF-κB to promote caspase-3 activation, OB may inhibit this activation and thereby shield cells from radiation-induced damage (Wang et al., 2022).

## 6 Interactions between PARP-1 and Caspase-3/AIF

**FIGURE 6:**
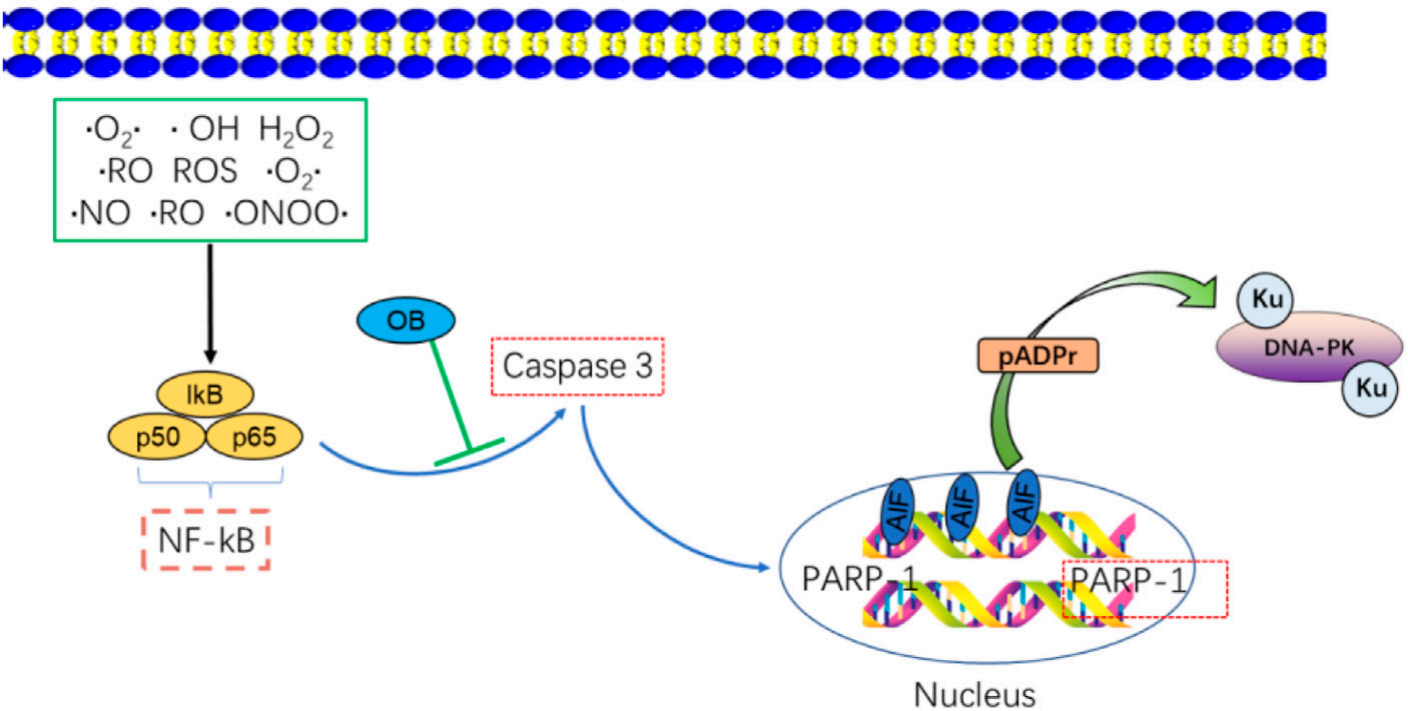
NF-κB activates the apoptosis-related molecule, caspase-3, OB inhibits caspase-3 activation by NF-κB/Caspase-3 activation resulting in the cleavage of PARP-1 and increased apoptosis. FRT may inhibit Bcl-2(Ca2+)/Caspase-3/PARP-1 signaling to reduce apoptosis in thymus cells.

Caspase-3 is a key effector caspase that triggers apoptotic cell death, partly through its ability to cleave substrate proteins, including PARP-1, DNA fragmentation factor (DFF45), and Lamin B (Boulares et al., 2001). Severe DNA damage and the consequent overactivation of PARP-1 can result in the excessive consumption of NAD+ and ATP energy stores. Caspase-3 activation can help preserve these energy supplies by cleaving the Asp-Glu-Val-Asp sequence in the PARP-1 nuclear localization signal, yielding two PARP-1 fragments (p89 and p24). The PARP-1 DNA binding domain is thus separated from its catalytic domain, inactivating its enzymatic activity. These p89 and p24 fragments can also serve as inhibitors of PARP-1 activity through monomeric poly aggregation and DNA binding, ultimately contributing to internucleosomal DNA degradation and eventual apoptotic death (Li and Darzynkiewicz, 2000). The positive feedback function of caspase-mediated PARP-1 inhibition underscores the critical role PARP-1 plays in apoptotic cell death (Figure 6).

Caspase-3 is a key protease that can cleave PARP and associated substrates, thereby facilitating the repair of DNA SSBs (Xu et al., 2016). High radiation levels can contribute to PARP-1 overactivation and consequent AIF release (Yu et al., 2006; Doti et al., 2014). Knocking down ATRX in the H460 lung cancer cell line promoted radiation-induced cell death, potentially through mechanisms linked to this PARP-1/Caspase-3 axis (Wieler et al., 2003). 5-azacyclobutane can enhance the expression of caspase-3 and the cleavage of PARP induced by ionizing radiation, thereby increasing the radiosensitivity of C666-1 nasopharyngeal carcinoma cells (Cai et al., 2017). HMGB1 silencing was associated with higher levels of Caspase-3, PARP, p-p38, and p-JNK proteins compared with radiation alone *in vitro,* suggesting that HMGB1 knockdown can promote caspase-3 upregulation and the cleavage of PARP. Increases in ROS production can promote DNA damage, increasing cell radiosensitivity (Han, 2016). Severe DNA damage can potentially trigger PARP-1 overactivation, which leads to the depletion of energy reservoirs NAD and ATP, and subsequently induces the translocation of AIF to the nucleus to facilitate caspase-independent apoptosis (Srinivasan, 1997). According to reports, the accumulation of PAR can trigger AIF-dependent cell death upon exposure to carbon ion radiation (Park et al., 2020). Poly (ADP- ribose) glycohydrolase (PARg) is a key enzyme important for PAR degradation, and the effects of PARg deficiencies on the sensitivity of murine embryonic stem (ES) cells to low and high LET radiation have been analyzed in prior reports. Increases in PARg-deficient ES cell sensitivity to γ-radiation are related to defective DSB repair and coincide with sensitivity to many other forms of radiation (Shirai et al., 2013). Combining plant-derived sphingosine and γ-radiation can enhance the death of radioresistant tumor cell lines through the nuclear translocation of AIF in response to ROS-mediated Bax relocation and the activation of PARP-1 independent of ROS. NAC is an antioxidant that can prevent AIF translocation by suppressing Bax relocation, although it does not suppress PARP-1 activation. AIF translocation is also reduced by pretreatment with the PARP-1 inhibitor DPQ(3, 4-dihydro-5)-[4-(1-pilon-butanoxy) -butanoxy]-1(2H)-isopentone or by PARP-1 knockdown (Park et al., 2005).

**FIGURE 7:**
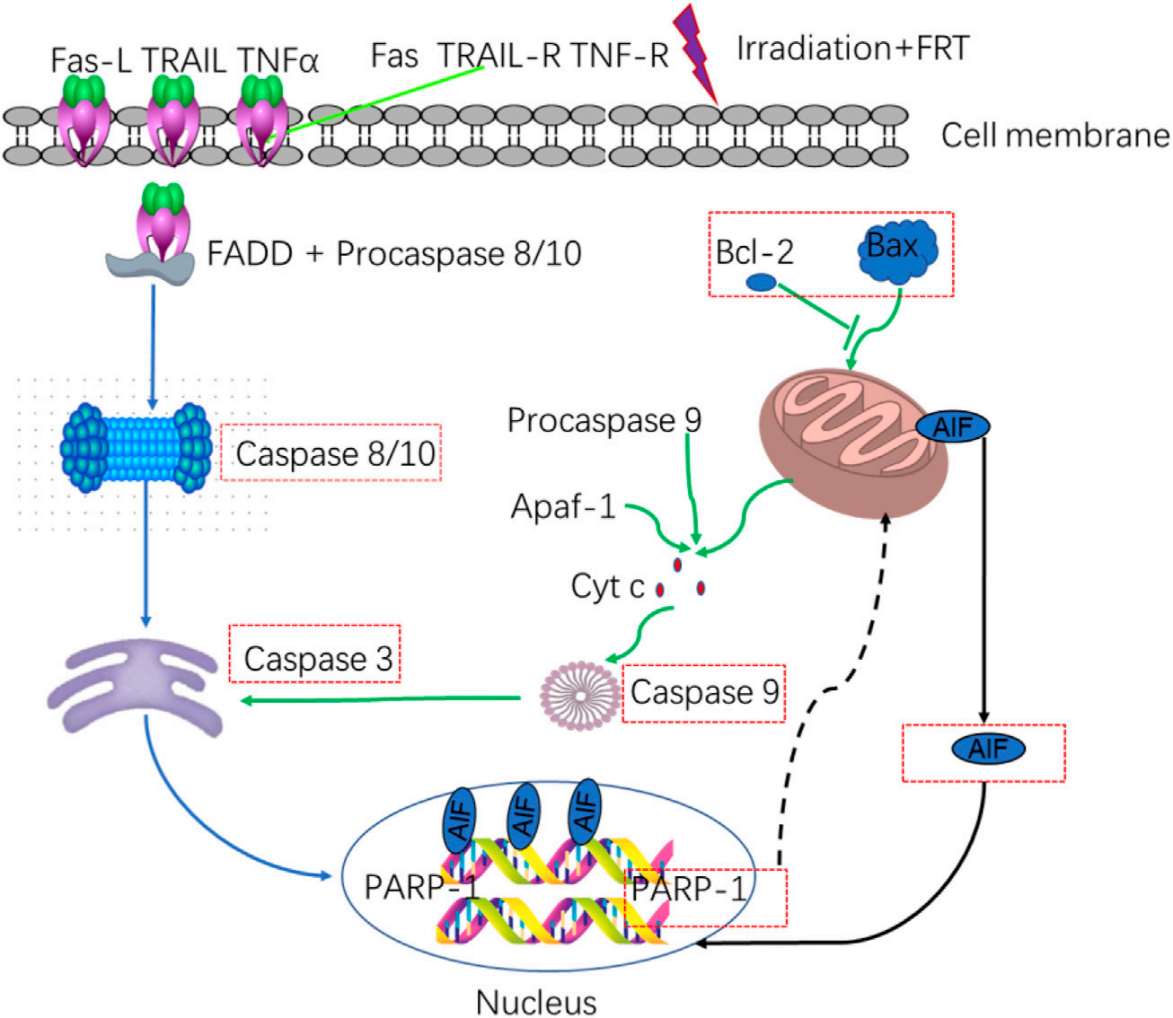
Relationship between apoptotic and inflammatory biomarkers. Biomarkers of apoptosis: caspases 3/8–10 and PARP-1/AIF.

FRT can significantly suppress the expression of pro-apoptotic factors such as caspase-3/8/9/10 and PARP-1/AIF-related signaling activity in thymus cells following radiation exposure, thereby enhancing radioprotective activity at least in part through the regulation of the PARP-1 and caspase-mediated pathways. FRT can also decrease the mRNA expression of several inflammatory factors, such as ICAM-1, VCAM-1, TNF-α, and NF-κB, without impacting the expression levels of IL-6 or IL-1α. It can also reduce the protein levels of ICAM-1, IL-1α, IL-6, TNF-α, and NF-κB after radiation exposure without affecting those of VCAM-1. As such, FRT may exert radioprotective efficacy by targeting these inflammatory pathways to reduce the inflammatory response (Figure 7) (Xu et al., 2018).

In summary, efforts to protect individuals from the damage associated with ionizing radiation exposure and to promote recovery following irradiation remain an area of active research interest. PARP-1 is a critical regulator of genomic integrity that facilitates DNA damage repair. Extensive DNA damage can result in PARP-1 overactivation, which can deplete cellular energy stores, culminating in cell death. Identifying mutations in genes involved in DNA repair may help guide the selection of appropriate candidates for PARP inhibitor treatment, informing clinicians as to whether these inhibitors should be provided as single-agent therapies or components of combination therapeutic regimens also incorporating inhibitors targeting other components of the DNA repair pathway, radiotherapy, or DNA damaging drugs. While many studies have explored PARP-1 as a target for radiosensitization in cancer treatment, considerably fewer have investigated FRT on normal healthy tissue. PARP-1 can function as a key modulator of cell fate and survival through its ability to control the activity of NF-κB, p53, and caspase-3/AIF (Figure 3). Although considerable progress has been made in understanding the relationship between PARP-1 and radiation sensitivity and in developing several PARP-1 inhibitors, the precise mechanisms connecting PARP-1 and radioprotection remain to be fully clarified. Therefore, further investigations are required to inform the design and selection of suitable radioprotective agents interacting with this pathway.

## 7 Opportunities and challenges of PARP inhibitor

Since its discovery, PARP-1 has been extensively characterized as a regulator of numerous biologically important pathways. PARP inhibitors provide new therapeutic opportunities to cancer patients, leading to their approval as single-agent treatments for certain cancer types. While the next-generation of these small-molecule inhibitors is in active development, the current versions continue to be applied as radiosensitizing drugs in clinical trials in combination with radiotherapy and chemotherapy. Further advancement in PARP inhibitor research could fully underscore the value of targeting this pathway for treating various diseases, thus providing novel therapeutic options to numerous patients, including those currently without effective treatments.

(1) Determining an optimal low-intensity dose regimen

While early experiments showed that single doses of Olaparib, Rucaparib, Niraparib, and Talazoparib were the maximum doses tolerated, it is unclear whether the maximum tolerated dose is appropriate for a tumor-selective drug that is not expected to damage normal tissues. Therefore, opting for the optimal biological dose instead of the maximum tolerated dose might be preferable to improve patients’ quality of life. Further research is needed to determine whether the low-intensity dose regimen, which may be less toxic and more affordable, is as effective as the approved single-dose regimen.

Toxicity issues and the need for meticulous titration of the cytotoxic agents and PARP inhibitors still constrain the combined use of PARP inhibitors with genotoxic chemotherapy and radiotherapy. A well-designed dose plan will be necessary to optimize the therapeutic index, which may have to be considered on an individual basis. The preclinical and clinical data show that when PARP inhibitors are combined with cytotoxic drugs, curative effects, and tolerance can be achieved with shorter treatment courses and lower dosages. We posit that it is necessary to reclassify PARP inhibitors based on their dual molecular mechanisms to accurately evaluate suitable combination strategies (Murai et al., 2012; Murai et al., 2014; Fojo and Bates, 2013).

(2) Identifying predictive biomarkers

A crucial objective, alongside determining the optimum tolerance for combined PARP inhibitor therapy, is the discovery of predictive biomarkers for patient segregation in conjunction with the therapy and understanding the impact of this combination on the resistance mechanisms against PARP inhibitors to achieve a more sustained clinical response. A critical query is whether a combined treatment regimen could alleviate the resistance to PARP inhibitor monotherapy seen in patients with BRCA mutations. It remains uncertain how much the combined administration of PARP inhibitors and other drugs can delay or prevent the onset of PARP inhibitor resistance in these patients and potentially lead to longer-lasting responses. Furthermore, the sequence and timing of administering the therapeutic agents demand careful consideration.

(3) Understanding the clinical mechanisms that drive resistance to PARP inhibitors in BRCA-mutant cancers

The prospect of mitigating resistance to PARP inhibitor monotherapy in patients with BRCA mutations through combination therapy presents an intriguing question. It is unclear whether the simultaneous use of PARP inhibitors and auxiliary agents could impede or prevent the emergence of resistance to PARP inhibitors in these patients, potentially resulting in more enduring responses. Furthermore, the arrangement and timing of administering these therapeutic agents call for meticulous planning and examination.

The presence of BRCA mutations does not universally predict the sensitivity of tumors to PARP inhibitors. In fact, PARP inhibitors display a spectrum of sensitivity in BRCA-mutant cancers. For instance, ovarian cancers with BRCA mutations show greater sensitivity than breast, prostate, or pancreatic cancers with the same mutations. Furthermore, cancers with BRCA mutations that are usually unrelated to BRCA carriers are even less sensitive to PARP inhibitors (Curtin et al., 2019; Jonsson et al., 2019). Genome screening techniques, like Myriad myChoice, may help identify tumors with defective homologous recombination but might produce false positives. These methods also detect genomic changes resulting from diminished HRR, which can persist even after HRR recovery (a phenomenon known as genomic scarring). This could result in failing to identify tumors where the HRD phenotype has been reversed and are resistant to PARP inhibitors. The most reliable predictive biomarker of HRD may be functional, such as the ability of tumor cells to form RAD51 lesions, which has been used to show that approximately one-third of abdominal tumors and high-frequency lung tumors contain homologous recombination defects (Patterson et al., 2014; Gentles et al., 2019).

(4) Leveraging biological insights into the response to PARP inhibitors to guide the design of combination therapy

Enhancing our understanding of the biological responses to PARP inhibitors could significantly contribute to designing effective combination therapies involving PARP inhibitors. Given that PARP inhibitors trigger extreme genomic instability, resulting in cancer cell death, one potential strategy could involve leveraging this phenotype. This could be achieved by pairing PARP inhibitors with agents that amplify cell death processes, such as apoptosis or necrosis, in the context of enhanced genomic instability (Yuan et al., 2013).

The research conducted over the past few decades indicates that the combined use of PARPi holds immense promise for cancer treatment, provided several significant hurdles can be overcome.

## Figures and Tables

**Table:** 

3-AB	3-aminobenzamide
ADP	Adenosine diphosphate
ADP-ribose	Adenosine diphosphate ribose
AIF	Apoptosis-inducing factor
AMPK	Adenosine 5‘-monophosphate (AMP)-activated protein kinase
ATF-4	Activating factor 4
BER	Base excision-repair
BMN	PARP-1/2 inhibitor Talazoparib (BMN-673)
BRCA	Breast-Cancer susceptibility gene
cNHEJ	Classical NHEJ
CypA	Cyclophilin A
DFF45	DNA fragmentation factor
DNA	Deoxyribonucleic acid
DNA-PK	DNA-dependent protein kinase
DPQ	4-Dihydro-5[4-(1-piperindinyl)butoxy]-1(2H)-isoquinoline
DSB	Double-stranded break
FRT	Flavonoids from *Rosa Roxburghii* Tratt
GPI 15427	10-(4-methyl-piperazin-1-ylmethyl)-2H-7-oxa-1,2-diaza-benzo[de]anthracen-3-one
HMGB1	High mobility group box 1
HPV	Human papilloma virus
HR	Homologous recombination
HRD	HR pathway deficiencies
HRR	Homologous recombination repair
HYDAMTIQ	2-dimethylaminomethyl-9-hydroxy-thieno[2,3-c] isoquinolin-5(4H)-one
IKK	IκB kinase
iNOS	Induced nitric oxide synthase
LET	Linear energy transfer
MMEJ	Microhomology-mediated end-joining
MMR	DNA mismatch repair
mTOR	Mechanistic target of rapamycin
NAC	n-acetylcysteine
NAD	Nicotinamide adenine dinucleotide
NER	Nucleotide excision repair
NF	Nuclear Factor
NHEJ	Non-homologous end joining
NHEJ	Non-homologous end joining
NMDA	N-methyl-D-aspartate
NMS-P118	1-(4,4-Difluorocyclohexyl)piperidin-4-yl]-6-fluoro-3-oxo-2,3-dihydro-1H-isoindole-4-carboxamide
OB	Onjisaponin B
PARg	Poly (ADP-ribose) glycohydrolase
PARP-1	Poly (ADP-ribose) polymerase 1
PDX	Patient-derived xenograft
ROS	Reactive oxygen species
SIRT1	Sirtuin 1
SMAD	Small mother against decapentaplegic
SRX	Synchrotron radiation X
SSB	Single-stranded break
STAT	Signal transducers and activators of transcription
TGF-β	Transforming growth factor-β1
XRCC1	X-ray repair cross complementing1
